# Modulation of vigilance/alertness using beta (30 Hz) transcranial alternating current stimulation

**DOI:** 10.3389/fnins.2025.1445006

**Published:** 2025-02-13

**Authors:** Zhongliang Chu, Rui Wang, Tianyi Zhou

**Affiliations:** ^1^Department of Psychology, Faculty of Arts and Sciences, Center for Cognition and Neuroergonomics, State Key Laboratory of Cognitive Neuroscience and Learning, Beijing Normal University, Zhuhai, China; ^2^State Key Laboratory of Cognitive Neuroscience and Learning, Beijing Normal University, Beijing, China; ^3^Beijing Key Laboratory of Applied Experimental Psychology, National Demonstration Center for Experimental Psychology Education (Beijing Normal University), Faculty of Psychology, Beijing Normal University, Beijing, China

**Keywords:** vigilance, tACS, brain stimulation, beta, compensatory tracking task (CTT)

## Abstract

Vigilance refers to the ability to maintain alertness and sustain attention for prolonged periods to detect and respond to subtle changes in the environment. Previous research has explored the use of transcranial alternating current stimulation (tACS) to modulate brain oscillations and enhance vigilance/alertness. In this study, we explore the modulation effects of different stimulation parameters on Vigilance using an open-source dataset. The open-source dataset includes within participant application of High-Definition tES (HD-tES) types, targeting two cortical regions (frontal, motor) with one stimulation waveforms (30 Hz); combining human-participant high-density electroencephalography (EEG) with continuous behavioral metrics. We only analyzed the behavioral task performance data to assess how vigilant states are acutely altered by specific tES types. Our findings indicate that (1) Both online and offline tACS improve vigilance performance; (2) online tACS have greater effect on vigilance performance than offline tACS; (3) tACS that targeting frontal region have greater effect on vigilance performance than stimulating the motor region. These results align with the view of current the theoretical accounts on the oscillatory nature of vigilance attention and contribute to the groundwork for tACS closed-loop interventions for counteracting vigilance decrements.

## 1 Introduction

Vigilance entails sustained attention to detect and appropriately respond to rare but critical changes over prolonged periods (Oatley, 1970). Many professions and academic tasks require individuals to maintain vigilance for extended periods. Research has consistently shown that individuals often experience a decline in vigilance over time, resulting in decreased performance in terms of response times and accuracy (van Schouwenburg et al., 2021; Warm et al., 2008; Robertson and O’Connell, 2010). Given the importance of vigilance, it is crucial to explore strategies to mitigate these decrements (Martínez-Pérez et al., 2022).

Neural oscillations, which are rhythmicity or repetitive neuronal activities in the central nervous system, are known to play a significant role in cognitive functions, particularly in vigilance and alertness (Coulborn et al., 2020). Researchers have turned to neuroimaging to better understand the neural mechanisms underlying vigilant attention (van Schouwenburg et al., 2021). A meta-analysis by Langner and Eickhoff (2013) identified a network of brain regions essential for maintaining vigilant attention, including the presupplementary motor area (pre-SMA), midcingulate cortex, anterior medial prefrontal cortex (PFC), mediolateral and ventrolateral PFC clusters, anterior insula, parietal areas, and subcortical structures. Furthermore, activity in the frontoparietal network has been associated with fluctuations in cognitive control necessary for vigilance. Functional brain imaging studies consistently show the involvement of frontal cortex activity in vigilance decrements.

Several electroencephalogram (EEG) studies have reported attention-related increases in the beta frequency band (13–30 Hz) (Makeig and Inlow, 1993; Al Qasem et al., 2022; Hsu and Jung, 2017). Makeig and Inlow (1993) introduced a vigilance annotation method known as local error rate. They computed the average coherence between slow fluctuations in EEG power and local error rate for each EEG frequency. They found a positive relationship between local error rate and EEG power in the alpha and delta frequencies, while noting a negative correlation at other frequencies (Makeig and Inlow, 1993). Furthermore, the exploration of frequency bands in the cortical structure suggests a potential link between the beta-band (13–30 Hz) and attentional function (Al Qasem et al., 2022). Hsu and Jung (2017) evaluated the information on alertness present in each subject’s complete EEG spectrum and arrived at similar conclusions to Makeig and Inlow (1993) and Al Qasem et al. (2022). In summary, beta oscillations appear to have a significant role in our capacity to sustain vigilance and alertness over time.

Transcranial electrical stimulation (tES) is a non-invasive neuromodulation technique that affects movement, mood, and cognitive function by delivering low-intensity electrical current to the scalp (Annarumma et al., 2018). The most well-known forms of tES include transcranial direct-current stimulation (tDCS), transcranial alternating-current stimulation (tACS), and transcranial random noise stimulation (tRNS) (Al Qasem et al., 2022). tACS entails the administration of alternating electric fields to the scalp, influencing specific oscillation frequencies in predetermined brain regions and modulating neural activity (Wischnewski et al., 2023). This process is thought to synchronize neurons in the underlying neural tissue with the stimulation frequency (Herrmann et al., 2013). Cognitive processes can be influenced by applying endogenous regional frequency currents linked to cognitive function.

In recent years, there has been a significant increase in research investigating the potential benefits of tACS by applying a weak alternating electric field during cognitive tasks (Dayan et al., 2013). One particular area of focus for tACS studies has been on vigilance/alertness behavioral metrics. Some researchers have observed a frequency-dependent effect on the vigilance decrement in tACS experiments (van Schouwenburg et al., 2021; Clayton et al., 2015; Rostami et al., 2021; Cui et al., 2018; Loffler et al., 2018). They suggested that tACS stimulation may enhance alertness. In Grover’s meta-analysis, the outcome within the cognitive domain indicated that tACS was beneficial for improving behavioral performance related to vigilant attention (Grover et al., 2023). Additionally, Martínez-Pérez et al. (2022) found that alpha tACS was more beneficial in ameliorating the effects of decrement in vigilance than theta tACS. van Schouwenburg et al. (2021) conducted a study where participants received theta and alpha tACS over medial PFC. They found frequency-dependent effect on the vigilance decrement over time became worse after theta compared with alpha stimulation. Rostami et al. (2021) also found that theta frequency (6 Hz) tACS over medial PFC was able to produce significant modulations in vigilant attention task. Furthermore, it modulated vigilant attention associated brain networks in healthy participants. Clayton et al. (2015) found that 2.0 mA of alpha-tACS (10 Hz) over occipitoparietal cortex prevented deterioration in two different vigilance tasks. The authors concluded that alpha oscillations promote top-down control processes and vigilance stability. While there is a range of findings on the effectiveness of tACS in enhancing vigilance performance, research on the impact of beta tACS on vigilance and alertness performance remains limited.

The tACS can be administered ‘online’ or ‘offline’. ‘Online’ tACS is the active tACS applied during cognitive tasks, whereas ‘offline’ tACS is administered immediately before or between tasks (Veniero et al., 2015), and it is more associated with changes in synaptic plasticity, rather than entrainment (Bland and Sale, 2019). There are more studies with measurements taken after tACS administration (‘offline’) than those (Grover et al., 2023) with behavioral measurements taken during tACS administration (‘online’). However, the effectiveness of ‘online’ versus ‘offline’ tACS patterns has not been extensively studied. Loffler et al. (2018) indicated that ‘online’ gamma-tACS can enhance performance in vigilance tasks by decreasing the slowdown of reaction times. Stecher et al. (2017) calculated the accuracy in the visual vigilance task over time and found that average accuracy in both ‘online’ and ‘offline’ alpha-tACS block were lower than baseline (before stimulation). However, Clayton et al. (2015) found that ‘online’ alpha-tACS over the occipitoparietal cortex prevented deterioration in vigilance tasks. In contrast, Cui et al. (2018) found the reaction time decreased over a period of time during ‘online’ 6 Hz tACS, while ‘offline’ 6 Hz tACS did not show a significant change. Further research is needed to determine if tACS in other frequency bands has similar effects. Overall, existing studies have not provided a clear consensus on the effects of ‘online’ versus ‘offline’ tACS patterns on sustained attention during vigilance tasks.

In studies involving non-invasive brain stimulation (NIBS), stimulation targets and parameters are typically selected based on known frequency bands and regions associated with sustained attention or previous research findings. While these approaches have shown some success, there is no guarantee that every parameter combination will effectively influence vigilance and alertness. This study aimed to investigate whether beta tACS could impact participants’ performance on cognitive tasks related to vigilance/alertness and identify the beta tACS montage that consistently improved sustained attention. Additionally, the study sought to determine the more effective pattern (‘online’ or ‘offline’) of tACS in counteracting vigilance decrements by exploring the effects of active tACS and the potential for aftereffects during the offline phase. The underlying hypothesis is that if such aftereffects exist, they may influence task performance in ways distinct from the immediate effects of online stimulation. To address these objectives, the study utilized the open-source dataset titled ‘Dataset of concurrent EEG, ECG, and behavior with multiple doses of transcranial electrical stimulation’ (Gebodh et al., 2021). This dataset combines high-density EEG data from human participants with physiological and continuous behavioral measurements during high-definition tES (HD-tES), providing a comprehensive dataset for analyzing the optimal stimulation montage for enhancing vigilance. The study focused on data from Experiment 2, where participants underwent two sessions involving frontal and motor stimulation, each with ‘online’ and ‘offline’ beta-tACS. The study defined the tACS on state as “online” and the tACS off state as “offline.” Behavioral data collected within the initial 20 min of the experiment served as baseline data. Online data was acquired during each stimulus trial. Behavioral data captured between the termination of the $i_{th}$ stimulus and the initiation of the subsequent i+1 stimulus was denoted as offline data. Comparing the mean and standard deviation of baseline, online, and offline behavioral data can help address the research question.

## 2 Materials and methods

### 2.1 Dataset description

The dataset for this analysis comes from a larger publicly available repository of EEG recordings, collected from a laboratory at The City University of New York. The dataset is one of the largest concurrent tES, EEG, Electrocardiogram (ECG), and behavioral datasets. It contains over 70 min of EEG data recorded during a continuous compensatory tracking task (CTT) developed by Makeig and Jolley (1995) and Huang et al. (2008). The data involved 20 neurologically typical individuals (7 females, 13 males) aged between 19 and 43 (mean age: 29.10 ± 6.75) recruited from the New York metropolitan area.

All experimental procedures were reviewed and approved by the Western Institutional Review Board and all procedures were conducted in accordance with the ethical guidelines set forth by the Declaration of Helsinki in 1964 and its later amendments. All participants were financially compensated for their participation. Data collection and procedures are detailed elsewhere (Gebodh et al., 2021) and is briefly detailed below.

The dataset was collected in two main experiments. The experiment 1 was a parameter space mapping experiment that explored different combinations of applying tES at different scalp locations and different stimulation frequencies. In total, experiment 1 explored 9 stimulation montages, which consisted of 3 scalp locations: Frontal, Motor, and Parietal; and 3 stimulation frequencies: 0, 5, and 30 Hz. Each stimulation montage was applied across 4 trials in each participant, with each bout of stimulation lasting 30 s with a 5 s ramp up and 5 s ramp down. In total, data from 10 participants was collected for Experiment 1, where the 9 stimulation montages were applied across three 70 min sessions (3 stimulation montages per session). At the conclusion of Experiment 1, the stimulation combinations of Frontal 30 Hz (F30) and Motor 30 Hz (M30) were selected as the best candidates to examine in Experiment 2.

**Figure 1:**
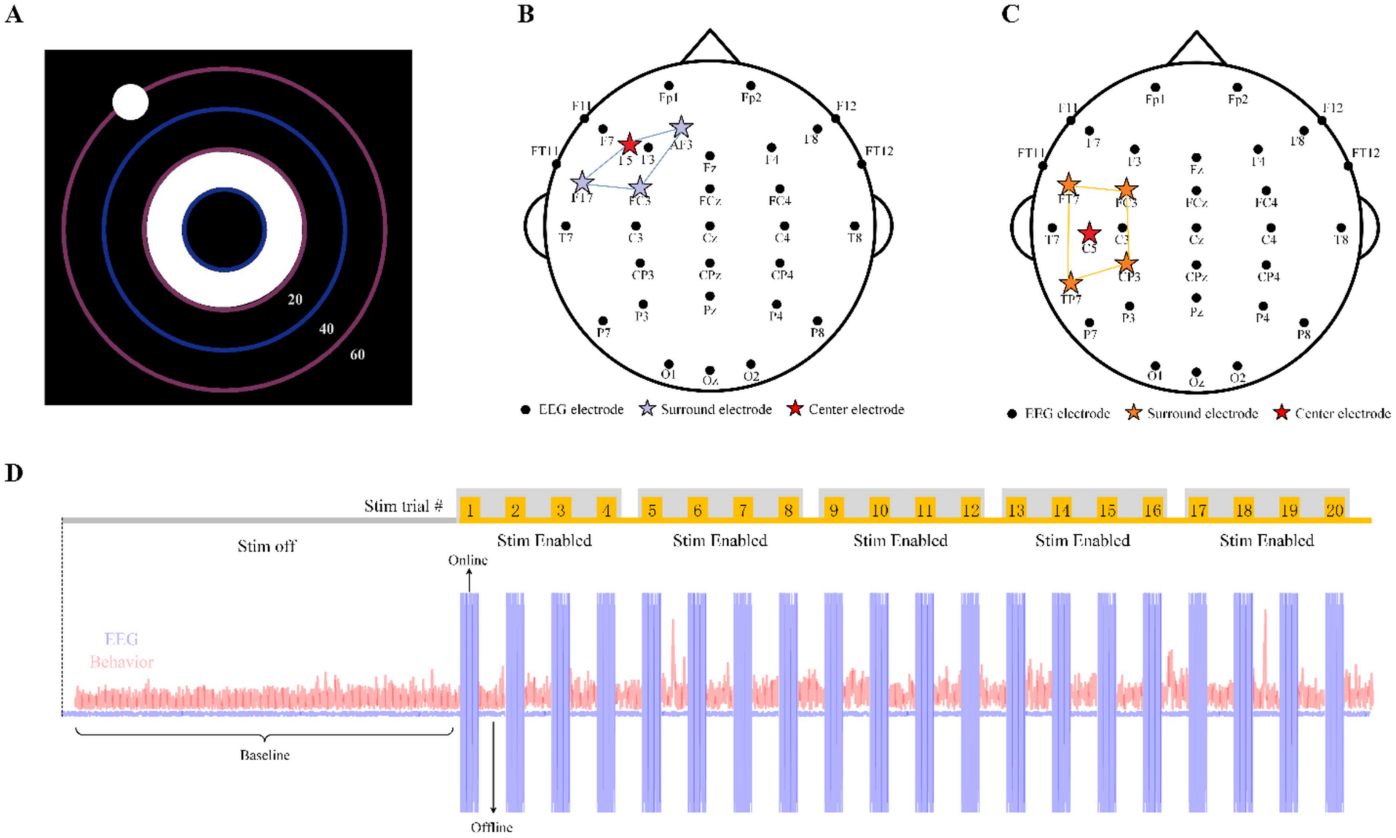
Behavioral task, EEG and stimulation montage. **(A)** The panel of CCT behavior task. **(B)** EEG and stimulation locations for F30. **(C)** EEG and stimulation locations for M30. **(D)** Block implementation with time series of a complete experimental series for exemplary participants (either M30 or F30).

For Experiment 2, F30 and M30 were applied to 16 participants over 20 trials (Figures 1B,C), where each trial consisted of 30 s of stimulation with a 5 s ramp up and 5 s ramp down. The application of these stimulation montages was broken up across two 70 min sessions, where one stimulation montage was applied per session. The author of the dataset provided 10 participants’ data for Experiment 1 and 15 participants’ data for Experiment 2. The study analyzed data of Experiment 2 and our research sample size is 15.

### 2.2 HD-tACS

HD-tACS was administered in 30-s epochs per trial, with an additional 5-s ramp-up/down period, at two different locations on the head using one stimulation waveform. The two stimulation conditions were defined by the area of stimulation (frontal or motor) and the frequency of the stimulation current (30 Hz). The conditions were represented by the first letter of the location followed by the frequency (e.g., frontal 30 Hz represented as F30). A total of 9 Ag/AgCl sintered ring stimulation electrodes were placed at standard EEG 10/10 locations to create two possible configurations of Nx1 HD-tACS, where *N* = 3 for frontal and *N* = 4 for motor stimulation. For each montage, N electrodes were selected for the outer (surround) ring electrode, and one electrode was selected as the center electrode. Note, some electrode locations were shared across montages with varied “ring” or “center” assignments. For frontal stimulation, surround electrodes (*N* = 3) were placed at AF3, FT7, FC3 and the return or center electrode was placed at F5 (Figure 1B). For motor stimulation, surround electrodes (*N* = 4) were placed at FT7, FC3, CP3, TP7, and the return or center electrode was placed at C5 (Figure 1C). All participants received 1 mA of stimulation (peak to peak).

### 2.3 Cognitive task paradigm and experimental procedure

Participants were instructed to use their mouse or pointing device to keep the ball near the target on the screen in a dark room. The ball moved automatically but was influenced by the participants’ mouse movements. They were asked to keep the ball in the center of the ring or as close as possible to the inner circle (Figure 1A). The deviation of the ball from the center of the target would be measured and reflect the participants’ performance in maintaining precise control and focused attention throughout the task. Prior to each session, participants engaged in a brief practice session lasting between 1 and 3 min. The session itself lasted 70 min, including a 20-min initial Stim Off period and a 50-min Stim Enabled period with 20 trials of stimulation. The CTT ran continuously and uninterrupted throughout both experiments. Participants were unaware of the block design and type of stimulation for each experiment.

As described in Figure 1D, each session (one for F30 and one for M30) included one stimulation off block (“baseline,” 20 min) followed by five consecutive stimulation enabled blocks (10 min each). In each session, either the F30 or M30 stimulation montage was randomly assigned to be administered during the five stimulation enabled blocks. The assigned stimulation montage consisted of 4 consecutive tACS stimulation trials during each block, totaling 20 trials of tACS stimulation per session in the experiment. Behavioral tasks were continuously performed, with tACS stimulation trials interleaved between the behavioral task trials. Current for each trial was ramped up over 5 s, sustained for 30 s at a maximum intensity of 1 mA (Max Stim Current), and then ramped back down. In the experiment of our research, behavioral task under stimulation refers to online tACS behavioral data. The rest of the time (no stimulation) evenly distributed between the stimulation trials. Behavioral task in these time slices refers to offline tACS behavioral data, which means offline tACS behavioral data is without the direct influence of tACS.

### 2.4 Data processing

The behavior data of experiment 2 was selected for further analysis in this study. Behavioral data (CTT circle deviation) were smoothed with a 5 s moving average window for then averaged for each trial. The calculated percent change in deviation between the during stimulation (Δ) and pre stimulation period was used as a marker of response to stimulation, whereas this same calculation for the stimulation off periods was used as marker of response to no stimulation. With this configuration a negative delta (−Δ) indicated that participants’ behavioral performance or response increased or got better with the given condition, whereas as a positive delta (+Δ) indicated that participants’ behavioral performance or response decreased or got worse with the given condition. Following this, the mean and standard deviation of the baseline, online, and offline behavioral data were calculated.

### 2.5 Statistical analysis

For baseline comparisons, non-parametric Wilcoxon signed-rank tests were applied. To evaluate the impact of tACS on vigilance behavior, group discrepancies in correct deviation or deviation variability were examined through a two-way ANOVA for repeated measures involving a stimulation montage factor (frontal vs. motor) and a stimulation pattern factor (‘online’ vs. ‘offline’). *Post hoc* two-tailed *t*-tests were conducted with Bonferroni’s correction in case of significant results in either the stimulation montage factor or stimulation pattern factor. A significance level of *p* < 0.05 was deemed significant.

## 3 Results

**Figure 2:**
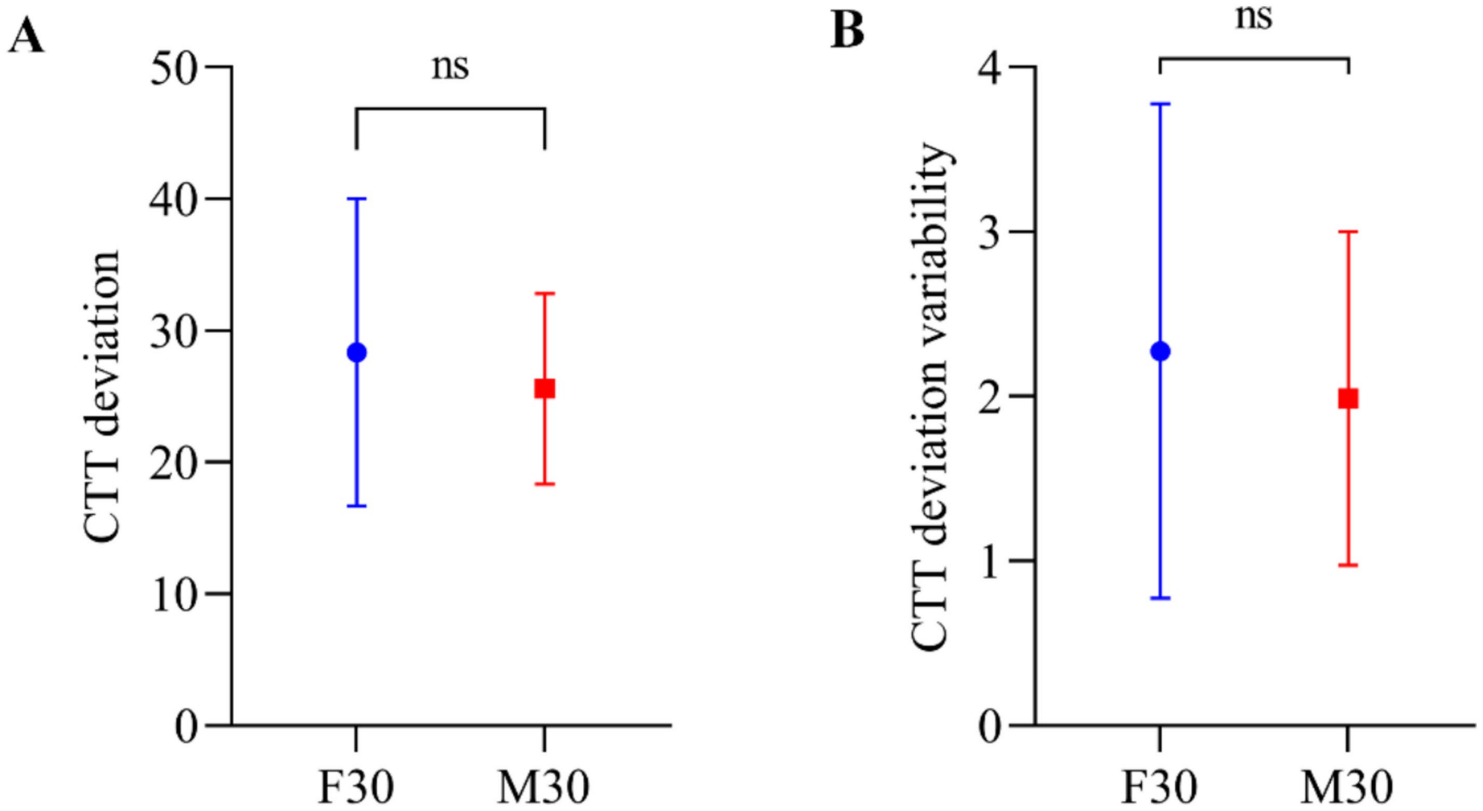
**(A)** CTT deviation in the CTT task. Error bars represent standard deviation of the mean. **(B)** CTT deviation variability of the participants’ mean deviation in the CTT task. Error bars represent standard deviation of the CTT deviation variability.

The study analyzed the mean values of baseline CTT deviation (during the initial 20 min of the session), with 28.37 for F30 and 25.59 for M30. Standard deviations of the baseline CTT deviation were calculated as 11.27 for F30 and 6.98 for M30. Additionally, the mean values of baseline CTT deviation variability were 2.28 for F30 and 1.99 for M30, with standard deviations of 1.45 for F30 and 0.98 for M30. The Wilcoxon signed-rank test was used to compare CTT deviation and CTT deviation variability between participants in the frontal tACS and motor tACS experiments. The results in Figure 2 showed no significant difference in baseline behavior data between F30 and M30 (*p* = 0.1688 for CTT deviation and *p* = 0.3591 for CTT deviation variability), indicated there was no performance difference before frontal or motor stimulation occurred.

**Figure 3:**
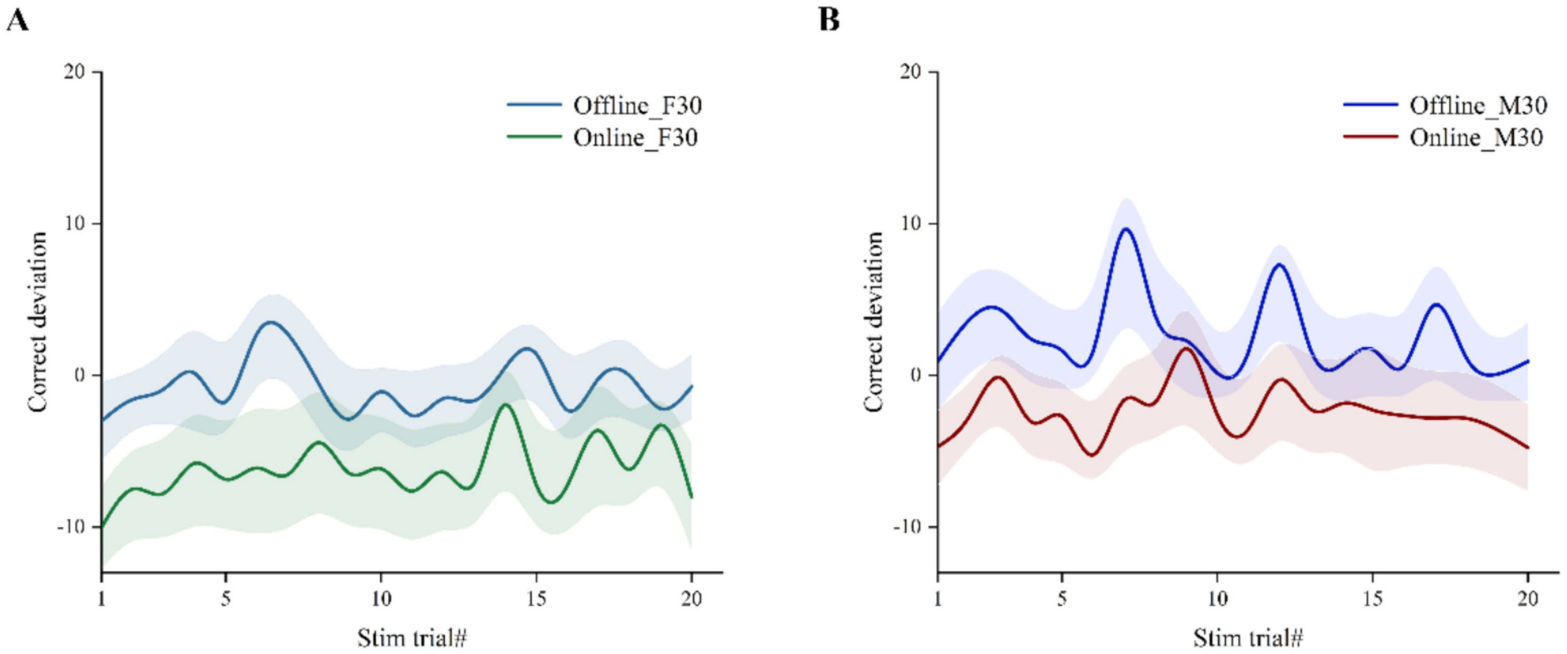
Correct deviation in CTT task during stimulation at each stim trial. Error bars with fill area represent standard error of the mean. **(A)** ‘Online’ vs. ‘offline’ in F30. **(B)** ‘Online’ vs. ‘offline’ in M30.

Overall, beta tACS significantly impacted performance in the CTT task compared to baseline, except for the ‘offline’ M30 condition. Figure 3 illustrates that ‘online’ data for both F30 and M30 had lower correct deviation than ‘offline’ data in each stim trial, indicating that ‘online’ tACS may enhance vigilance/alertness performance more effectively.

**Figure 4:**
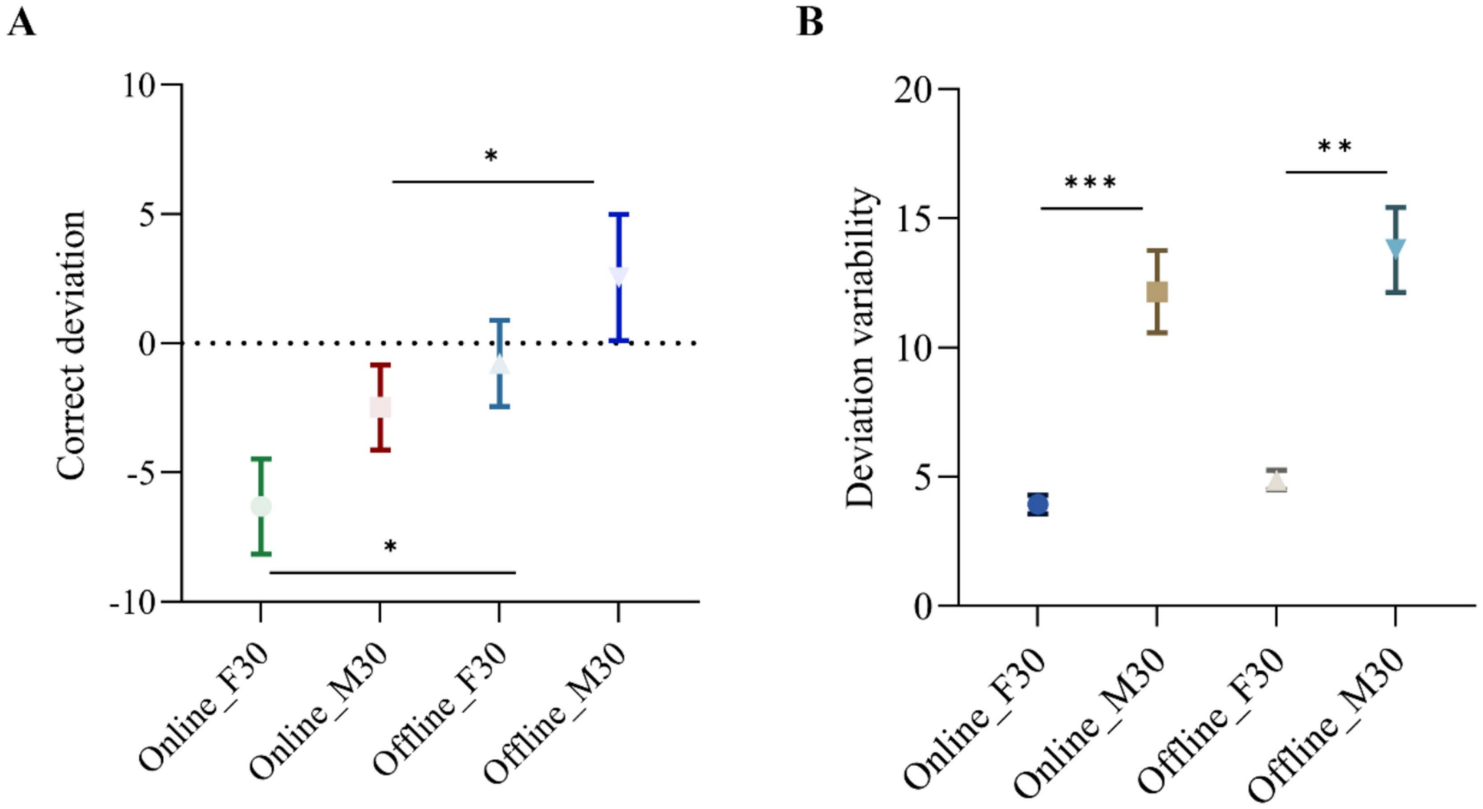
**(A)** Correct deviation for ‘online’ F30, ‘online’ M30, ‘offline’ F30 and ‘offline’ M30 among participants (*N* = 15). ∗ = *p* < 0.05. Error bars represent standard deviation of the mean. ∗∗ = *p* < 0.01. Error bars represent standard deviation of the mean. **(B)** Deviation variability of participants’ deviation changes for ‘online’ F30, ‘online’ M30, ‘offline’ F30 and ‘offline’ M30 among participants (*N* = 15). ∗∗∗ = *p* < 0.0005. Error bars represent standard deviation of deviation variability.

The correct deviation of the CTT task was analyzed through a two-way ANOVA with two factors: stimulation montage (frontal or motor) and stimulation pattern (‘online’ or ‘offline’). The main effect of stimulation pattern (‘online’ or ‘offline’) was found to be significant (*p* = 0.0099), indicating an improvement in CTT task performance when participants received stimulation (‘online’ tACS). Furthermore, a significant effect of stimulation montages was observed (*p* = 0.0140), showing a greater decrease in correct deviation of the CTT task with F30 compared to M30. The interaction effect between stimulation pattern and stimulation montage was not significant (*p* = 0.7611). The impact of stimulation pattern (‘online’ or ‘offline’) was also examined using Sidak multiple comparison tests in both frontal and motor areas (Figure 4A). It was significant in frontal areas (*p* = 0.0245) with a correct deviation of −7.124 and a 95% Confidence interval of (−13.29, −0.9615), as well as in motor areas (*p* = 0.0415) with a correct deviation of −6.511 and a 95% Confidence interval of (−12.77, −0.2556).

The deviation variability of the CTT task was also analyzed through a two-way ANOVA with two between-subject factors: stimulus montages (frontal or motor) and stimulus pattern (‘online’ or ‘offline’). As shown in Figure 4B, the main effect of stimulus pattern was significant (*p* = 0.0073) in terms of deviation variability in the CTT task, demonstrating the reliable effectiveness of ‘online’ tACS in enhancing vigilance/alertness performance. The main effect of stimulus montages in terms of deviation variability was also significant (*p* = 0.0004), indicating that deviation variability of the CTT task is smaller with F30 and larger with M30, highlighting the reliable effectiveness of frontal beta tACS in improving vigilance/alertness performance. The interaction effect between stimulation pattern and stimulation montage is not significant (*p* = 0.2937). The effect of stimulus montages (‘frontal’ or ‘motor’) was further examined using Sidak multiple comparison tests in two stimulus patterns (‘online’ or ‘offline’). The results were significant for ‘online’ pattern (*p* = 0.0005) with correct deviation values of −8.818 and a 95% Confidence interval of (−13.13, −4.509), as well as ‘offline’ pattern (*p* = 0.0016) with correct deviation values of −9.789 and a 95% Confidence interval of (−15.31, −4.267).

Our results of the tACS effect in the CTT task revealed three key findings. Firstly, there was a main effect of stimulation pattern (‘online’ versus ‘offline’), indicating that ‘online’ tACS in frontal and motor areas resulted in higher vigilance/alertness performance compared to ‘offline’ tACS. Secondly, we observed an influence of stimulation montage (frontal versus motor), with frontal stimulation having a more significant impact on modifying vigilance/alertness. Specifically, ‘online’ beta tACS over the frontal area showed the most pronounced moderation effect on vigilance performance among ‘online’_F30, ‘online’_M30, ‘offline’_F30, and ‘offline’_M30. Lastly, we found that 30 Hz tACS on the motor (‘offline’) area contributed to vigilance decrements. Our discussion delves into these findings in relation to existing literature, offering potential explanations for the results and suggesting areas for further research.

## 4 Discussion

The application of transcranial alternating current stimulation (tACS) for modulating cognitive function has sparked significant interest, yet the existing literature presents inconsistent findings. This study aimed to investigate the effects of various stimulation targets and parameters on tACS to pinpoint the most promising avenues for future research. Specifically, we sought to assess the efficacy of beta tACS on the frontal region compared to the motor region in modulating vigilance/alertness performance. Additionally, we examined the differences between ‘online’ and ‘offline’ stimulation. Our analysis utilized the publicly available dataset ‘Dataset of concurrent EEG, ECG, and behavior with multiple doses of transcranial electrical stimulation’ (Gebodh et al., 2021). This dataset contains comprehensive electroencephalography (EEG) data from human participants along with physiological and continuous behavioral measurements during transcranial electrical stimulation (tES). We focused on the data from Experiment 2. Mean and standard deviation values of participants’ behavioral data were compared for online_F30, online_M30, offline_F30, and offline_M30 among the 15 participants to evaluate the effects of beta-tACS modulation on vigilance/alertness performance.

Stimulation of the frontal areas has a significant effect on improving vigilance levels, while stimulation of the motor areas may not effectively prevent vigilance decline. Chen et al. (2020) used simultaneous EEG-fMRI to explore the connection between vigilance and brain activity, finding that participants with stronger vigilance displayed increased activity in the frontoparietal network (FPN). Our research indicates that tACS applied to the left frontal areas enhances alertness. Moreover, Wang et al. (2022) discovered that frontal tACS at a stimulation frequency of 30 Hz had a drowsiness-preventing effect on participants. This aligns with our own findings that frontal stimulation enhances vigilance levels. The motor cortex plays a crucial role in observing and imagining actions, as well as executing skilled limb movements (Vargas-Irwin et al., 2018). Our study demonstrated that ‘online’ motor stimulation enhances movement control and execution, thus preventing vigilance decrement. However, the ‘offline’ motor tACS led to vigilance decrements in our results. The larger standard deviation of the participants’ behavioral data in the ‘offline’ motor stimulation compared to frontal stimulation also indicated the instability of vigilance levels in the ‘offline’ session. These results suggest that the effect of enhancing vigilance performance with ‘offline’ motor tACS was significantly less effective than that of frontal stimulation.

Online beta-tACS demonstrates superior modulation of vigilance attention compared to offline beta-tACS. Entrainment of a significant number of neurons occurs consistently during online tACS stimulation, particularly when electric field values exceed 0.3–0.4 mV/mm. Stronger electric fields recruit more neurons and impact cognition and behavior (Wischnewski and Schutter, 2017). Entrainment is likely responsible for the increased regularity in spike timing observed (Vossen et al., 2015). Our results show that the mean correct behavior of participants during online tACS stimulation (F30 and M30) is lower than the mean correct behavior of participants without stimulation (offline tACS in F30 and M30). Additionally, the standard deviation of participants ‘correct behavioral data in each block of online tACS is smaller compared to offline, indicating greater reliability in alertness level during online stimulation. These findings suggest that beta-tACS is more effective in enhancing vigilance levels through entrainment and increased regularity in spike timing compared to offline effects (short-term aftereffect).

Recent studies highlight non-invasive brain stimulation methods as promising countermeasures against vigilance decrements, surpassing traditional methods like caffeine intake or nootropic drugs. Our study confirms the efficacy of tACS in improving vigilance and supports this notion. However, most vigilance studies, including ours, have primarily focused on behavioral measures. Future research should aim to address this gap by investigating the physiological mechanisms underlying the activating effects of these stimulation protocols.

While some meta-analyses have suggested that cognitive function improvements are typically more pronounced after completing tACS (‘offline’) rather than during tACS stimulation (‘online’) (Grover et al., 2023), our findings differ from this trend. This discrepancy may be attributed to the inconsistencies in experimental conditions (such as stimulus montage and frequency) and outcomes, which have made it challenging to definitively establish a direct correlation between the ‘online’ and ‘offline’ effects across stimulation conditions. In our study, we conducted a clear comparison between ‘online’ tACS and ‘offline’ tACS, and concluded that ‘online’ tACS (30 Hz HD-tACS) applied to frontal and motor areas resulted in higher vigilance performance compared to ‘offline’ tACS.

However, it is important to note that our study had limitations, including a relatively small sample size per group and the restriction of the sample to normal individuals, which limits the generalizability of our findings to those with pathological vigilance deficits. Although the use of public datasets enhances the reproducibility and comparability of our research, the limitations of the dataset (such as sample size and variable range) may have impacted our results. What’s more, Future studies could explore alternative datasets or design more detailed experiments to provide a more comprehensive understanding of these issues. In this study, the dataset used only included stimuli presented on the left side, which may introduce a lateralized bias in the interpretation of the results. Future studies should incorporate stimuli presented on both sides to verify the generalizability of the findings and explore potential functional differences between hemispheres.

A growing body of research suggests that abnormal brain oscillations are a common feature of neurological and mental disorders. To further our understanding of how rhythmic non-invasive brain stimulation (NIBS) techniques can modulate brain activity, future studies should explore the connection between real-time (‘online’) and delayed (‘offline’) effects. Researchers should also pay attention to the duration of these effects in future experiments.

Frequency-tuned non-invasive brain stimulation (NIBS) involves matching the frequency of an externally applied entraining stimulus with naturally occurring oscillations (Vosskuhl et al., 2018). The synchronization of ongoing oscillations is most effective when the frequencies of the entraining stimulus align with the endogenous frequencies (Ali et al., 2013). Additionally, most previous studies and the dataset we used have neglected both the fact that vigilance comprises two dissociable components (i.e., arousal and executive vigilance) and the potential role of differences in arousal levels. In vigilance tasks, there were three key factors regarding sustained attention: the multicomponent nature of vigilance, the potential role of individual differences in arousal level at baseline and the oscillatory nature of sustained attention (Martínez-Pérez et al., 2022). Future research should focus on personalized stimulation frequencies and the various aspects of vigilance, while also taking into account individual differences in baseline arousal levels to potentially improve the effectiveness of interventions.

## 5 Conclusion

While there is mounting evidence that tACS allows to regulate vigilance and alertness performance during (online to) stimulation, we find mixed evidence for the usage of stimulation targets and parameters. The study aimed to figure out whether beta tACS would positively affect performance on participants’ cognitive tasks of vigilance/alertness and determine the more effective pattern (‘online’ or ‘offline’) of tACS. The result indicated that employing tACS in the frontal region had a significant effect on cognitive tasks that assess vigilance/alertness. ‘Online’ tACS over frontal region led to higher vigilance/alertness level compared with online tACS over motor region and offline tACS over frontal and motor.

## Data Availability

Publicly available datasets were analyzed in this study. This data can be found: https://openneuro.org/datasets/ds003670/versions/1.1.0.
